# Salvage Total Hip Replacement for Failed Proximal Femoral Fracture Fixation

**DOI:** 10.7759/cureus.74613

**Published:** 2024-11-27

**Authors:** Zain Habib, Aditya Malik, Matthew Moran, Muhammad Umer Rasool, Mohammed Arifuzaman, Azeem Ahmed, Rama Mohan

**Affiliations:** 1 Trauma and Orthopaedics, Manchester University NHS Foundation Trust, Manchester, GBR; 2 Trauma and Orthopaedics, North Manchester General Hospital, Manchester, GBR; 3 Intensive Care Unit, North Manchester General Hospital, Manchester, GBR; 4 General Surgery, North Manchester General Hospital, Manchester, GBR

**Keywords:** complex arthroplasty, hip revision arthroplasty, long-term clinical outcomes, radiological outcomes, revision arthroplasty

## Abstract

Introduction: Salvage arthroplasty for failed proximal femoral fracture fixation is a complex procedure. This involves the removal of the primary failed or broken implant followed by a hip joint replacement procedure. The complications and technical difficulties associated with these surgeries are often difficult to anticipate.

Methods: Initially, to further understand the position in the literature with regard to salvage arthroplasty, we completed an informative scoping review. Search terms were selected, and databases Embase and PubMed were utilised to form a literature search. Relevant articles were selected by two independent researchers, with a final list of nine studies reviewed and tabulated for themes to be identified and analysed. Subsequently, we retrospectively studied the notes of all the patients who underwent complex conversion arthroplasty in the same district hospitals in a span of 16 years (August 2002 to August 2018) and presented the results.

Results: Seventy-one patients underwent complex salvage arthroplasty following a failed fixation of proximal femoral fracture under the care of three different surgeons. All surgeries were carried out by the posterior approach. The demographics, intraoperative events, and postoperative follow-up have been presented through clinical and radiological assessments. With a mean age being 73.6, female patients were almost twice the number of male patients. The left hip was the more common surgical site. Implant cutout was the most common cause of failure of the primary implant. Most of these surgeries were either uncemented (31 cases, 43.66%) or hybrid (29 cases, 40.84%). The most common acetabular size to be used was 50 mm, and the most common head size used was 32 mm. A majority of the surgeries were metal on poly bearings (64 cases, 90.14%). The mean surgical time, including anesthetic, was four hours and 13 minutes. A total of 31 (44%) patients needed blood transfusion postoperatively. The infection rate was 21.13% (15 cases), being the most common surgical complication. The mean follow-up of the patients was 27.2 months with the maximum follow-up being 125 months. The one-year mortality was found to be 14% (10 cases). The mean limb length discrepancy was shortening by an average of 3.84 mm. A total of 66.2% (47 cases) of patients were shortened postoperatively. The average cup abduction and anteversion angles were 35° and 24.25°, respectively. The average position of the femoral stem was 0.31° in the varus with 40.85% (29 cases) of patients having a slightly varus stem.

Conclusion: Upon drawing comparisons with primary hip arthroplasty for primary osteoarthritis through data available in the literature, it is obvious that salvage arthroplasty is a complex procedure with longer surgical times and onerous rehabilitation. Whilst it is not the same as revision arthroplasty, many of the characteristics in terms of surgical planning and outcomes are similar. Therefore, it is our recommendation that salvage hip arthroplasty procedures should be categorised, listed, and studied separately from primary arthroplasty in the National Joint Registry database.

## Introduction

Between 2020 and 2023, over 245,000 primary hip arthroplasties were performed in the UK [1]. Indication for surgery in 91% of these cases was for osteoarthritis without distinction between primary and secondary osteoarthritis and with no record of the complexity of the procedure. 

Salvage procedures for failed proximal femoral fracture fixation are often more challenging and complicated than total hip arthroplasty for primary osteoarthritis due to factors such as heterotopic ossification, femoral deformity, acetabular protrusion, bone defects, previous infection, retained metalwork, and cut-out implants [2,3]. This makes it a more challenging reconstructive procedure with a higher rate of complications [4]. These include increased risk of infection, dislocation, heterotopic ossification, sciatic nerve palsy, and intraoperative blood loss [5-8].

Due to these factors and complications of reconstructive surgery, it has been associated with a significantly higher morbidity when compared to total hip replacement for primary osteoarthritis [4]. Given that there are an estimated 72,000 hip fractures a year in the UK [9], the number of salvage procedures for failed proximal femoral fracture fixation is also on the rise [10]. It is, therefore, important that we understand and classify these fractures appropriately. 

## Materials and methods

Literature review

A scoping review of the literature was completed to ascertain the overall impression of the evidence with regard to salvage procedures for failed proximal femoral fracture fixation. Search terms such as 'conversion total hip arthroplasty', 'posttraumatic total hip arthroplasty', 'posttraumatic hip arthritis', 'salvage hip arthroplasty', and 'complications of conversion arthroplasty' were selected, and PubMed and Embase were used to produce a literature search. Relevant articles were selected by two independent researchers, with a final list of 12 studies reviewed. Studies pertaining to posttraumatic acetabular deformities were excluded as the focus of this study was on salvage procedures for failed proximal femoral fracture fixation. Ultimately, nine studies were analysed. Table 1 shows the summary of the selected studies with details of study design, patient demographic data, salient themes, and analysis.

**Table 1:** Tabulated data for scoping review of the literature on conversion arthroplasty THA: total hip arthroplasty

Citation information	Study design	Comments and analysis
Hernandez et al., 2018 [10]	A retrospective analysis between 2006 and 2014 of 64 consecutive patients undergoing conversion total hip arthroplasty after non-union of femoral neck fractures fixed percutaneously with screws	Five-year survival rate of 97% following the revision surgery. Concluded that conversion total hip replacement in their cohort was associated with low complication rates, excellent implant durability, and overall positive clinical outcomes. The caveat was that their patients had suffered minimally displaced/undisplaced fractures with low energy mechanisms. This led to the relative preservation of soft tissues resulting in a lower complication rate
Pui et al., 2013 [11]	A comparative retrospective study of conversions to total hip replacement between dynamic hip screws and intramedullary nails	The study observed a high rate of complications (both overall and orthopaedic) in the group with nails (overall 41.9% vs. 11.7% and orthopaedic 29.0% vs. 5.5%)
Baghoolizadeh et al., 2016 [2]	A retrospective analysis between 2009 and 2014, reviewing 2009 conversion hips vs. 69,868 primary hips	The study found that there was an increased risk of complications, surgical site infections, unplanned intubations, blood transfusions, sepsis, and an increased length of stay in the former group. No data was provided on functional outcomes in the long run however
Qin et al., 2018 [12]	A retrospective analysis from 2005 to 2014, reviewing 2145 conversion hips to 12666 primary hips	A higher rate of complications and increased length of stay in the conversion group. A higher rate of non-home-bound discharges was also found in the conversion arthroplasty group
McLawhorn et al., 2018 [13]	A retrospective analysis between 2007 and 2014, reviewing 2018 conversion hip arthroplasties, matched against 2018 primary hip replacements	There were increased complication rates, length of stay, operative times, non-home discharges as well as higher blood transfusion requirements in conversion arthroplasty patients as compared to the primary hip arthroplasty group
Archibeck et al., 2013 [14]	A retrospective analysis between Feb 1987 and October 2008 reviewing 102 conversion arthroplasties, including operations following 39 intertrochanteric fractures and 63 intracapsular fractures	They reported four periprosthetic fractures (3.9%), and all of them were in the group of patients having intertrochanteric fractures before their conversion arthroplasty. They concluded that there is an increased risk of such periprosthetic fractures as well as postoperative dislocation rate for conversion arthroplasty of failed internal fixation
McKinley et al., 2002 [15]	A matched-pair case-control study design was completed with two groups of patients between the ages of 60-80. Group 1 were 107 patients who had undergone salvage arthroplasties following failed fixation. Group 2 were 214 patients undergoing primary arthroplasty for neck-of-femur fracture	Group 1 displayed a complication rate of 36% postoperatively within one year. Out of these, revision was required for 17.8% due to the complications. Group 1 had a greater risk of early dislocation (odds ratio: 2.33; 95% CI: 10.2-5.32), infection (odds ratio: 4.0; 95% CI: 1.07-14.93), and overall complication (odds ratio: 2.78; 95% CI: 1.50-5.55). They also found a worse survival rate for Group 1
Lu et al., 2019 [3]	This was a review article delving into the literature of indications, preoperative planning, different techniques review, and postoperative care. Conversion arthroplasties were also discussed including the difficulties faced	They have summarised that intraoperative and postoperative complication rate is higher in conversion arthroplasty and hence requires meticulous pre op planning, intra op preparation and postop care
Mahmoud et al., 2016 [16]	A systematic review completed compliant with the Preferred Reporting Items for Systematic Reviews and Meta-analysis (PRISMA) system, using PubMed, Cochrane, and Embase. They carried out a systematic review to compare the outcomes of salvage THA and primary THA for intracapsular fractures of the femoral neck	The study found that salvage THA carries a significantly higher risk of complications than primary THA for intracapsular fractured neck of femur, including deep infection and early dislocation

Hernandez et al. reviewed 64 consecutive patients undergoing a conversion arthroplasty, reporting generally positive clinical outcomes for their patients when comparing to the limited data available in the literature on conversion arthroplasty and not primary arthroplasty [10]. However, the study only focused on conversion arthroplasty for failure of previous in situ dynamic hip screw/cannulated screw fixation of minimally and undisplaced femoral neck fractures, rather than for a more inclusive indication such as failure of previous intramedullary nail fixation. Fittingly, Pui et al., in a retrospective study, compared complication rates of conversion arthroplasty patients based on indications of failed intramedullary nail vs. failed dynamic hip screw fixation, finding that the nail group had a much higher overall complication rates, 41.9% vs. 11.7%, respectively [11]. 

Baghoolizadeh and Schwarzkopf [2], Qin et al., and McLawhorn et al. were comparative retrospective studies on clinical outcomes of conversion hip arthroplasty vs. primary hip arthroplasty between years 2009-2014, 2005-2014, and 2007-2014, respectively [12,13]. McLawhorn et al. managed to analyse exactly 2018 cases in each patient cohort [13]. All three studies found an increased rate of complication rate of various descriptions along with longer hospital stay in the conversion arthroplasty group. Archibeck et al. retrospectively reviewed 102 conversion arthroplasty cases from 1987 to 2008, concluding that these patients have a high risk of periprosthetic fractures and dislocation rates [14].

McKinley and Robinson completed a matched-pair case-control study between a conversion arthroplasty for failed fixation vs. primary arthroplasty in the hip fracture group for patients aged 60-80. They found an increased rate of complications along with higher mortality in the conversion arthroplasty cohort [15]. Lu and Phillips described, from the findings of a detailed review article, the pertinence of operative planning for conversion arthroplasty cases, due to their propensity for significant operative complications [3]. Finally, Mahmoud et al., a systematic review included in our literature review, compared outcomes between conversion salvage total hip arthroplasty vs. primary arthroplasty for hip fracture. They found a significantly higher complication rate in the former group, involving early dislocation and deep infection rates [16]. 

Majority of the studies were retrospective. This can be attributed to the paucity of such surgeries performed as well as the protracted recovery time. The patient cohort for salvage arthroplasties tends to be elderly and comorbid, thus precluding the possibility of long-term follow-ups in many cases, making it difficult to obtain level one evidence. 

The biggest weakness of the single available systematic review and meta-analysis is the inclusion of retrospective cohort studies out of pure necessity due to the lack of level one studies. The systematic review article also comments on the lack of a well-designed study; the ideal scenario would be a prospective, adequately powered, multi-centre randomised trial [16]. 

Aims and objectives

The primary objective of this study was to assess the clinical and radiological results following salvage procedures for failed proximal femoral fracture fixation. We aimed to compare the results with that of revision arthroplasty as well as primary arthroplasty for osteoarthritis without metalwork in situ. Based on these comparisons, recommendations for categorisation can be made, as they are currently categorised as primary arthroplasty in the National Joint Registry. 

Methodology

The study design and proposal for this study were approved by the Northern Care Alliance NHS Trust. As this study is a service evaluation carried out retrospectively with no direct patient contact, there was no requirement for ethical clearance or an informed consent process. Data handling protocols were followed meticulously to be in line with the National Institute for Health and Care Excellence (NICE) guidelines. This included limiting access to confidential information to relevant authors only. A list of patients who had undergone salvage procedures for failed proximal femoral fracture fixation were drafted by retrospectively reviewing logbooks of three prominent revision hip surgeons from August 2002 to August 2018. 

The inclusion criteria for patients were as follows: (a) prior proximal femoral fracture, (b) fixed with an implant (cannulated screws, dynamic hip screws, short femoral nails, proximal femoral locking plates), and (c) conversion to a total hip replacement following the failure of reconstruction. 

Neck-of-femur fractures treated with hemiarthroplasties were naturally excluded from our study, as this would fall into the category of revision surgery as opposed to ‘salvage’. Patients with previous acetabular trauma requiring conversion arthroplasties were also excluded as per the aim of this study. Patients with incomplete notes were also excluded.

The following parameters were evaluated from the notes: (1) age and sex of patient at the time of surgery, (2) side of surgery, (3) cause for failure, (4) surgical approach, (5) duration of surgery including anaesthetic time, (6) intraoperative blood loss and transfusion requirements (provided data was available), (7) postoperative blood transfusion requirements, (8) information on the cementation of implants, (9) implant type (including cup and head size) and bearings, (10) duration of hospital stay, (11) discharge destination, (12) intra- and postoperative complications (medical complications like anaemia, chest infection, acute kidney injury (AKI), etc., and surgical complications like infection, periprosthetic fractures, dislocations, aseptic loosening, and revision surgery), (13) one-year mortality, and (14) maximum hospital follow-up (orthopaedic and non-orthopaedic) 

All the available preoperative and postoperative radiographs of the patients were evaluated and analysed using TRAUMACAD software (Brainlab; Munich, Germany) for the following 10 parameters: (1) preoperative Dorr classification of the femur, (2) preoperative limb length discrepancy, (3) postoperative femoral offset, (4) postoperative acetabular offset, (5) postoperative global offset, (6) postoperative limb length discrepancy, (7) cup abduction angle, (8) cup anteversion, (9) quality of cementation of cup and stem (if applicable), and (10) stem varus/valgus angle.

Radiographic evaluation was carried out using the postoperative evaluation tool software TRAUMACAD, including measuring limb length discrepancy with ischial tuberosities and lesser trochanters as reference points. The X-rays on evaluation were also calibrated to 120% zoom in the routine anteroposterior (AP) pelvis X-ray, following advice sought from our radiology department.

## Results

Demography

A total of 71 patients were found that underwent conversion arthroplasty of the hip following a failed fixation of proximal femur fracture, including femoral neck, intertrochanteric, and subtrochanteric fractures (Table 2). 

**Table 2:** Demographics results table

Demographic variable	Recorded value
Mean age-years (range)	73.6 (52-94)
Female gender n (%)	47 (66.45%)
Male gender n (%)	24 (33.55%)
Affected side left n (%)	40 (56.25%)
Affected side right n (%)	31 (43.75%)

Clinical results

The aetiology of implant failure, thus requiring salvage arthroplasty, is depicted in Table 3. The majority were from implant screw cut-outs from the femoral head or failure of the implant following a non-union of the fracture.

**Table 3:** Indication for salvage procedure

Indication for salvage procedure	Frequency (n = 71)
Implant cut-out from the head n (%)	33 (46.48%)
Implant failure/break and non-union n (%)	21 (29.58%)
Secondary osteoarthritis n (%)	8 (11.27%)
Avascular necrosis of the femoral head n (%)	7 (9.86%)
Traumatic peri-implant fracture n(%)	2 (2.82%)

From the review of the notes, all 71 patients underwent a standardised operative approach. All patients underwent the posterior approach to the hip through a scar tissue where possible, with removal of the fixation device followed by preparation of the acetabulum and femur for implants, respectively. All patients had an induction antibiotic in the form of teicoplanin 400 mg, which was continued for 48 hours postoperatively or until samples were negative. A total of 15 patients (21.12%) amongst the 71 operated showed positive culture results for an infective organism. Six cases were subclinical and did not require any treatment. Eight cases were treated with suppression antibiotics therapy. One case was treated with a two-stage revision. The types of arthroplasty received by patients are presented in Table 4, implant size in Table 5, and surface bearing in Table 6. The majority received either uncemented or hybrid implants.

**Table 4:** Types of arthroplasty

Types of arthroplasty	Frequency (n = 71)
Cemented n (%)	9 (12.68%)
Uncemented n (%)	31 (43.66%)
Hybrid n (%)	29 (40.84%)
Reverse hybrid n (%)	2 (2.82%)

**Table 5:** Implant size range

Implant type	Range of size mm (mode)
Acetabular cup	44-58 (most common 50 mm)
Head	22-36 (most common 32 mm)

**Table 6:** Surface bearing

Surface bearing combination types	Frequency (n = 71)
Metal on metal n (%)	0
Metal on poly n (%)	64 (90.14%)
Ceramic on poly n (%)	7 (9.86%)

No significant complications were documented. Seven (9.86%) patients required intraoperative blood transfusion, with an average of 2.45 units per patient. One patient was set up with intraoperative cell salvage and autotransfusion. The surgical time ranged from 149 to 368 minutes (mean 253 minutes) from the induction of anaesthetic to the transfer to recovery.

A total of 44% (31 cases) of patients received blood transfusion postoperatively. Number of units required postoperatively ranged from one to five units (mean 2.55 units). All patients received chemical thromboprophylaxis and those patients who were on warfarin or direct oral anticoagulants (DOACs) were recommenced on these 48 hours postsurgery. Recorded postoperative parameters are collated in Table 7. 

**Table 7:** Postoperative parameter recordings DVT: deep vein thrombosis *Postoperative implant-related clinical parameters

Parameter n (%)	Frequency (n = 71)
Postop blood requirements n (%)	31 (43.75%)
Infection n (%)	15 (21.13%)
Subclinical Infection n (%)	6 (8.45%)
DVT n (%)	2 (2.82%)
Anaemia n (%)	9 (12.68%)
Trochanteric pain syndrome n (%)	7 (9.86%)
Poor mobility n (%)	7 (9.86%)
Postop pneumonia n (%)	2 (2.82%)
Acute kidney injury n (%)	2 (2.82%)
Electrolyte imbalance n (%)	4 (5.63%)
Haematoma n (%)	2 (2.82%)
Discharge away from home n (%)	13 (18.31%)
Dislocation n (%)*	0
Revision surgery n (%)*	1 (1.41%)
Sciatic nerve palsy n (%)*	1 (1.41%)
Periprosthetic fracture n (%)*	7 (9.86%)

The postoperative complications of the study group were recorded from their inpatient stay as well as outpatient follow-up. Length of hospital stay ranged from two to 78 days, with a mean of 34 days, longer than routine arthroplasty patients. No patients were lost to follow-up. Follow-up ranged from 12 to 125 months (mean 27.2 months). Any medical average follow-up was 47.87 months. One-year mortality for the study group was 14% (10 cases). 

Radiology outcomes

TRAUMACAD software was instrumental in assessing pre- and postoperative X-rays. The preoperative X-rays were evaluated for limb length discrepancy and the Dorr type of the femur based on the appearance of the trabecular and cortical bone for operative planning [17]. The mean preoperative shortening of the affected side was noted to be 13.87 mm, with a range of 0-40 mm. A total of 49.3% (35 cases) of the patients were a Dorr type B femur. The results are tabulated in Table 8.

**Table 8:** Preoperative radiograph analysis Recorded Dorr type of femurs

Dorr type	Frequency (n = 71) n (%)
A	7 (9.86%)
B	35 (49.3%)
C	29 (40.85%)

Postoperative parameters that were evaluated were limb length discrepancy, femoral offset, the medial offset (acetabular offset), and the global offset of the reconstructed hip (Table 9).

**Table 9:** Postoperative radiograph analysis 1 Recorded mean limb length discrepancy of the affected side Recorded femoral, acetabular, and global offset

Parameter	Mean value mm (range)
Limb length discrepancy of affected side	3.84 mm short (15 mm short to 15 mm long)
Femoral offset	39.22 (10-64)
Acetabular offset	25.78 (17-31)
Global offset	65.31 (47-93)

Moreover, the femoral component was analysed for its positioning with respect to the anatomical axis of the femur, that is varus, valgus, or neutral positioning. Furthermore, the cup abduction angle and the cup anteversion angles were measured (Table 10). The mean cup abduction angle was noted to be 35.06°, and the mean acetabular cup anteversion angle was noted to be 24.25°. 

**Table 10:** Postoperative radiograph analysis 2 Recorded femoral component malposition, acetabular cup abduction, and anteversion angle

Parameter	Mean value degrees ° (range)
Femoral component malposition	0.31° varus (7° varus to 5° valgus)
Acetabular cup abduction angle	35.06° (25°-49°)
Acetabular cup anteversion angle	24.25° (8°-36°)

The mean limb length discrepancy was towards a shortening of 3.84 mm, with the most significant shortening of 15 mm in a patient preoperatively which was 40 mm short. Despite this, no patients raised this as a complaint during follow-up. Further details of the average values of lengthened and shortened patients is depicted in Table 11.

**Table 11:** Postoperative radiograph analysis 3 Recorded types of limb length discrepancy

Type of limb length discrepancy	Frequency n (%) (n = 71)	Average value mm (range)
Lengthening	22 (31%)	7.40 (2-15)
Shortening	47 (66.2%)	5.09 (1-15)
Neutral	2 (2.82%)	N/A

The postoperative femoral component position was also assessed and recorded. The overall mean position of the stem was noted to be 0.31° of the varus. Further information is collated in Table 12.

**Table 12:** Postoperative radiograph analysis 4 Recorded values of femoral component stem malposition

Femoral component stem malposition	Frequency n (%) (n = 71)
Neutral	24 (33.8%)
Varus	29 (40.85%)
Valgus	18 (25.35%)

## Discussion

Preparatory to completing this study, the authors embarked on a scoping review of the literature. This review emphasised the lack of a well-designed study that conclusively commented on the outcomes of salvage procedures for failed proximal femoral fracture fixation. None of the studies reviewed took into account radiological parameters, such as stem and cup positioning. The data and analysis from this review of the literature seemed to depict that salvage procedures for a failed proximal femoral fracture fixation are in fact complex procedures, with complication rates, length of surgery, and hospital stay duration similar to that of revision arthroplasty, rather than arthroplasty performed for primary osteoarthritis. 

The reoperation rate following fixation of proximal femur fractures, due to any cause, is reported to be 0.5%-2.0% [18]. In elderly and frail patients, salvage total hip arthroplasty is more suitable to circumvent the complications of non-weight-bearing rehabilitation and the operative complications and jeopardy of internal fixation with osteoporotic bone [19]. Screw purchase is poor in osteoporotic bone, and thread cut-out is likely [20]. With the mean age of patients in our patient group being 73.6 years, salvage arthroplasty is a justified approach. More than 90% (64 cases) of our patients also had a Dorr canal type B and C, which further justifies the role of arthroplasty rather than internal fixation. 

Implant failure and cut-out formed the majority of the indications for salvage arthroplasty in our cohort, naturally leading to complex operations due to the residual bone defects in the femur. This can lead to the requirement for unconventional implants. In our study, it was found that a wide variety of implants were used to circumvent the possible complications of a long and demanding salvage arthroplasty surgery, including long uncemented revision stems, modular anatomical stems, and long cemented stems. Dual mobility cups and larger head sizes were used to provide more stability as a trade-off for increased wear in the long run. Efforts to reduce the risk of dislocation are relevant to salvage arthroplasty for implant failure, as loss of abductor attachment and redundancy of soft tissue can further complicate procedures [18]. Moreover, other challenges faced include difficult dissection due to postoperative scarring and adhesions, alterations in proximal femoral anatomy, and osteoporosis leading to poor bone quality and occurring from prolonged prior immobilisation [21,22].

The mean theatre time for our cohort was four hours and 13 minutes, including the anaesthetic time. As per the NHS website, typical hip arthroplasty can vary between one and two hours. This indicates that primary arthroplasty takes significantly less time than salvage arthroplasty, albeit our operative time is not being recorded separately. Due to the longer surgical times, it is also expected that oozing of the blood from the surgical field would be more significant than arthroplasty for a native hip. In our case series, it was observed that 9.86% (seven cases) of the patients required blood transfusion in the form of packed red blood cell units intraoperatively, and 44% (31 cases) of these patients required blood transfusion postoperatively. Comparatively less, Pederson et al. in a large population based follow-up study of 28,087 primary hip arthroplasties noted that only 32.3% of the patients received at least one unit of blood transfusion within the first eight days of surgery [23]. Postoperative patients receiving blood transfusions are at a higher risk of complications such as cerebrovascular accidents and pneumonia, with a higher 90-day mortality [23]. Consequently, the higher level of transfusion-related morbidity with salvage arthroplasties adds another dimension of complexity for patients, when comparing this procedure with primary hip arthroplasty.

Another debilitating postoperative complication, posing risk to life and limb is infection. Our study depicted an infection rate of 21.13% with 15 patients in total suffering with postoperative infection, albeit six being subclinical. Similarly, Gittings et al. retrospectively reviewed 33 patients in a single hospital that underwent salvage arthroplasty of the hip and reported an incidence of infection at 18% [24]. This is significantly higher than the rates of postoperative infection for uncomplicated primary arthroplasty, which have been reported to be as low as 1.3% [25]. With the life changing sequelae that an infection can cause in this cohort of patients, it is imperative that subclinical infection is ruled out preoperatively. This can be done using hip aspiration to confirm suspicion of infection amidst a background of other supportive factors such as clinical features and raised inflammatory markers [26].

In our case series, it was also noted that the mean time of admission following surgery was 35 days, in comparison to the 2-3 days till discharge following elective uncomplicated total hip replacement [25]. This delay in discharge for salvage arthroplasty indicates the further meticulous postoperative care and rehabilitation required in this patient cohort. Despite this, currently for the National Joint Registry, they are included in the category of primary hip replacements. However, it is clear from this study that conversion arthroplasties exhibit challenges similar to revision hip surgeries, including higher surgical times, increased need for blood transfusion, difficult surgical dissection, variability of implants, difficult rehabilitation, and longer admission times. An initiative to force salvage arthroplasties as their own entity would facilitate improved research on a national scale, leading to the development of an evidence-based and streamlined management approach to these patients [4]. Naturally, this would also allow for improved resource utilisation as researchers grasp an understanding of the fiscal implications of salvage arthroplasties on hospitals and institutions. 

The radiological parameters observed in this study provide a useful tool to reflect the results of salvage arthroplasties, albeit length measurements only being accurate within a correction of 20% as a result of zoom calibration. Parameters with acetabular cup angle, however, provide a more reliable analysis. Many surgeons in the past have studied and described a safe range that provides optimal stability and reduces the risk of hip dislocation [27,28]. Abduction and anteversion are key components of this with consensus being that safe ranges to prevent dislocation are 30°-50° and 20°-40°, respectively. Muscle imbalances are also an important contributor to dislocations [29]. In our study, the majority of the acetabular implants lay in the safe zone suggested, with zero dislocations at follow-up. Only one patient (1.4%) underwent a revision surgery due to infection, rather than dislocation or implant failure. 

In our follow-up, seven patients (9.86%) represented with periprosthetic fractures. Unsurprisingly, this is closer to the value of the Mayo Clinic Joint Registry for periprosthetic fractures in revision hip surgery of 4% from a total of 6349 cases than it is to the 1.1% of periprosthetic fractures in primary hip arthroplasty [30].

Whilst only being a retrospective analysis, leading to the risk of recall bias, of a small group of patients, this is the first study available in the literature that has taken into account both the clinical and radiological parameters. Moreover, it is not a cohort study where we can accurately draw comparisons from a similar group of matched patients that underwent total hip arthroplasty for primary arthroplasty. We have, however, drawn some comparisons from similar studies available in literature. Due to the same reason, we have simply presented our findings as observed. It is a level four evidence study, inferior to the evidence gleaned from prospective trials, with all data being collected from a single NHS centre in a limited case series, with the potential for confounding factors that are difficult to assess and control. 

## Conclusions

The results from this study and the available literature demonstrate that salvage arthroplasty for failed proximal femoral fracture fixation is a complex procedure requiring meticulous surgical planning and expertise. Generally, it is a successful surgical procedure but has a higher rate of limb and life-threatening complications including prosthetic joint infections, periprosthetic fractures, and intraoperative blood loss. As a result, salvage hip arthroplasties are more similar in characteristics to revision total hip arthroplasty rather than primary total hip replacement.

It is, therefore, our recommendation that salvage arthroplasty be reclassified as a distinct and separate category from primary hip arthroplasty in the National Joint Registry. This would improve data collection and our understanding of these surgeries on a national scale to improve patient outcomes and cost containment and aid the development of a streamlined management approach.
